# Supplementary material for: Insulin/IGF-1-mediated longevity is marked by reduced protein metabolism
Source: Mol Syst Biol. 2013 Jul 2;9:679. doi: 10.1038/msb.2013.35 (PMC3734508; doi:10.1038/msb.2013.35)

Supplementary Information belonging to:

# Insulin/IGF-1 mediated longevity is marked by reduced protein metabolism

By G.J. Stout *et al.*

**Table of content**:

Supplementary Figure legends

Supplementary Table legends

Supplementary Figures S1- S11:

S1: Mass spectral analysis

S2: Peptide correlation analysis

S3: Protein localization analysis

S4: Distribution of protein abundance

S5: ORA of enriched biological pathways

S6: Comparative analysis with published datasets

S7: Duplex proteomics experiment

S8: Western analysis of ribosomal proteins

S9: Brood size experiment

S10 : Polyribosome profiling

S11: mRNA analysis

## SUPPLEMENTARY FIGURE LEGENDS

**Figure S1:** Mass spectral analysis. Representative mass spectrum of an identified and quantified peptide derived from RPL-17 by mass spectrometry, identified after trypsin digestion, TMT labeling and SCX fractionation of pooled extracts. Sequence and MS/MS HCD Spectrum of the RPL-17 peptide (aa 89-98) as identified by MASCOT software (See material and methods) are shown. Identified b and y ions are indicated. The relative abundance of RPL-17 in each of the three strains was determined in duplicate from the low mass range spectrum magnified in the inset, indicating reduced abundance of RPL-17 in *daf-2(e1370)* nematodes.

**Figure S2:** Peptide correlation analysis. Correlation between the peptides quantified for each unique mass tag in the 6-plex TMT experiment. Relative intensities of the identified peptides for biological duplicates of each of the three tested strains (upper panels) as well as the correlation between the strains (bottom panels): N2 (126 and 127), *daf-2(e1370)* mutants (128 and 129) and *daf-16(mu86); daf-2(e1370)* mutants (130 and 131). *r* indicates Pearson Correlation coefficient and orange reference line indicates perfect correlation $r = 1$.

**Figure S3:** Protein localization analysis. Localization and expression of the differential expressed proteins between *daf-2(e1370)* and N2 proteomes. The observed proteins with differential abundance are expressed in **a**) all cellular compartments and **b**) in a broad range of organs and tissues throughout the nematode.

**Figure S4:** Multiple proteomics changes between *daf-2(e1370)* and N2 nematodes. Histogram showing distribution of protein abundance ratios between synchronized *daf-2(e1370)* and N2 nematodes at day one adulthood. The cut-off for increased (yellow) and decreased (blue) abundance was set at 30% compared to N2. FC: Fold Change.

**Figure S5:** ORA of enriched biological pathways in the *daf-2(e1370)* mutant. Pathway analysis using ORA identified numerous significantly increased (yellow) and decreased (blue) processes in the *daf-2(e1370)* mutants as compared to N2. For complete list we refer to **Suppl. Table I** and **II**, respectively.

**Figure S6:** Comparative analysis with published microarray and proteomics studies. **a)** Overlap between *daf-2* specific differentially expressed genes and proteins as specified by Murphy *et al.* (Murphy et al, 2003) or Dong *et al.* (Dong et al, 2007), respectively, and the quantitative proteomics dataset presented here. “Other” indicates genes and proteins that were differentially expressed in the indicated studies respectively, but were found unchanged in the here presented proteomics data set. **b)** Direction of regulation of overlapping genes from (a) is consistent with our quantitative proteomics dataset as determined by GSEA. *p* - value was calculated using a Hypergeometric distribution test using 18,000 *C. elegans* genes as background.

**Figure S7:** Independent proteomic analysis of *daf-2(e1370)* and N2 nematodes. An independent biological replicate of N2 and *daf-2(e1370)* was performed using duplex TMT LC-MS/MS analysis. **a**) Overlap between 6-plex and duplex datasets. **b**) Strong correlation between proteins identified in 6-plex (y-axis) and duplex (x-axis). Dashed line

indicates perfect correlation ($r = 1$), **c**) Comparative analysis of the direction of changes in protein abundance between 6-plex and duplex analysis.

**Figure S8:** Western analysis of ribosomal proteins. Protein extracts for each strain were analyzed by Western blotting using the indicated antibodies detecting proteasome 20 α-subunits (20Sα), ribosomal proteins L22 and L28 and Ureb1 which detects E3 ligase EEL-1. Actin was used to normalize protein abundance intensities. Numbers below each blot represent quantification by densitometry of the protein intensities relative to N2.

**Figure S9:** Brood size experiments were performed with indicated mutant nematode strains at 25°C. The average amount of eggs for at least 15 nematodes per strains is presented as mean ± S.E.M. Statistical differences were calculated Student t-test with Welch correction (*** $p < 0.001$; ** < 0.01).

**Figure S10**: Polyribosome profiles were performed with indicated nematode strains at 15°C (a) and 20°C (b). Average peak height is determined in at least 3 independent biological replicates and presented as mean ± S.E.M. Statistical differences were calculated Student t-test with Welch correction (* $p < 0.05$).

**Figure S11**: Total mRNA was extracted from 1500 worms at indicated temperatures. The *daf-2(e1370)* mutant contained less mRNA than N2 when propagated at 20°C. mRNA yield (ng/µl) was determined for each strain in triplicate, using poly(A)$^+$ trapping. Statistical differences were calculated Student t-test with Welch correction.

## Supplementary Table Legends

**Table I**: ORA results for proteins with increased abundance in *daf-2* proteome.

**Table II**: ORA results for proteins with decreased abundance in *daf-2* proteome.

**Table III**: GSEA results for enriched processes and pathways.

**Table IV**: Overview of lifespan data of tested strains (A) and upon siRNA mediated targeting (B). *p* – values indicate the statistical differences between the tested lifespan and the corresponding control (indicated by a number in column 4). The median lifespan is expressed as a percentage of control median lifespan (last column, control set to 100%).

**Table V**: Overview of lifespan data in *daf-16(mu86)* background (A) and upon siRNA-mediated targeting (B) *p* – values indicate the statistical differences between the tested lifespan and the corresponding control (indicated by a number in column 4). The median lifespan is expressed as a percentage of control median lifespan (last column, control set to 100%).

**Table VI**: Overview of performed brood size experiments of indicated strains (A), and upon siRNA mediated targeting (B). The brood size is expressed as a percentage of control (set to 100%).

Stout et al, Supplementary Figure S1

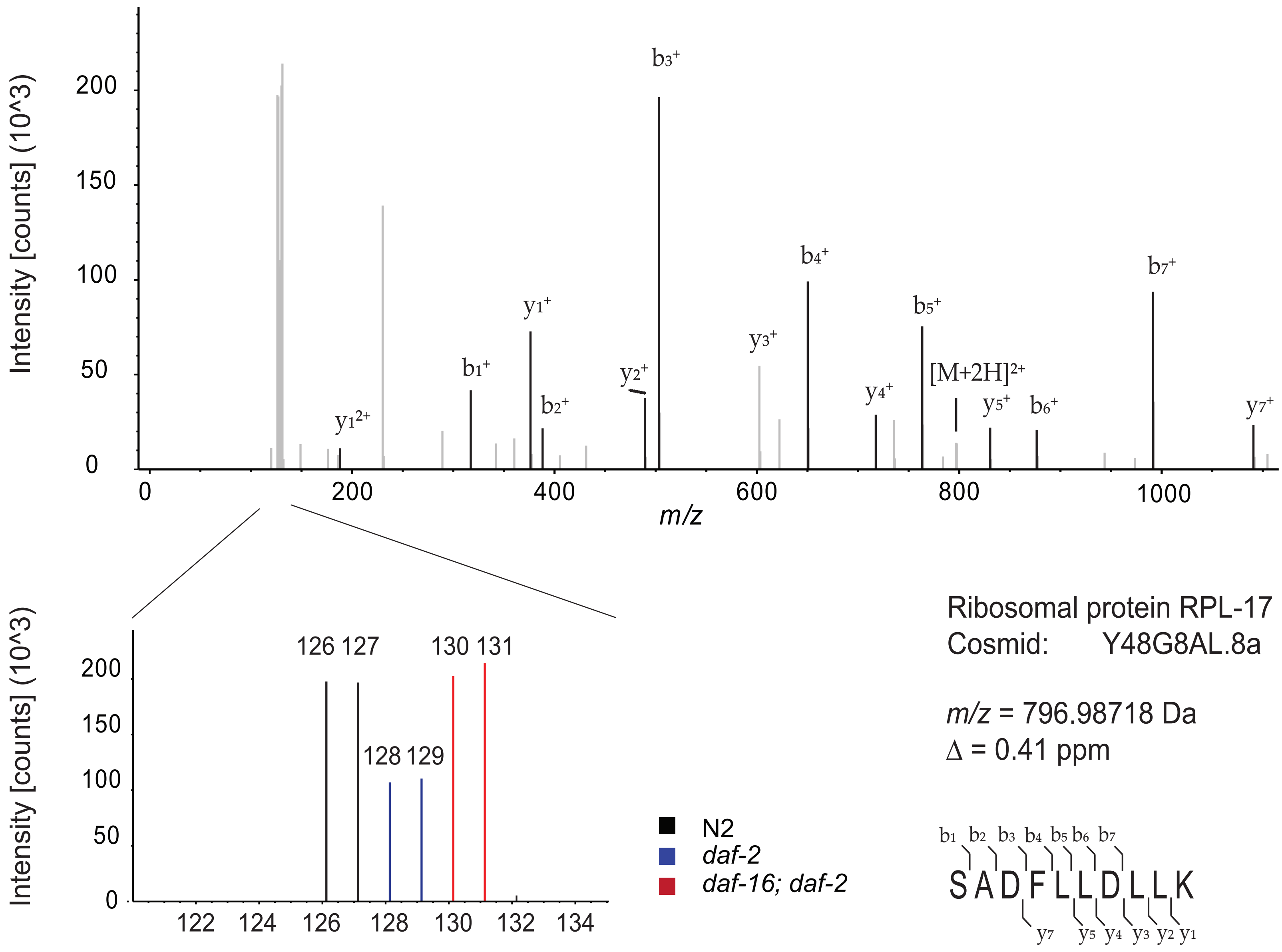

Stout et al, Supplementary Figure S2

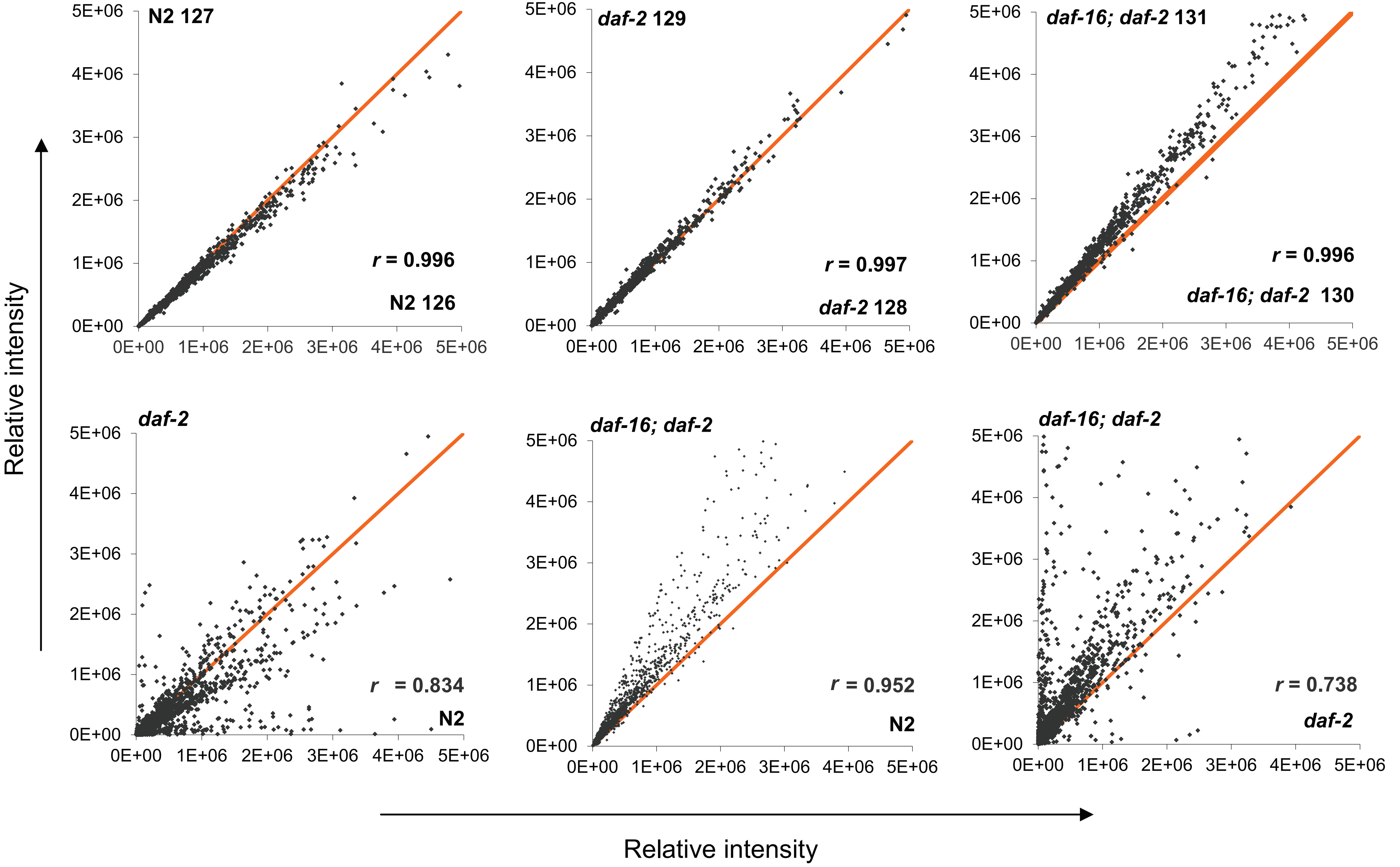

Stout et al, Supplementary Figure S3

a

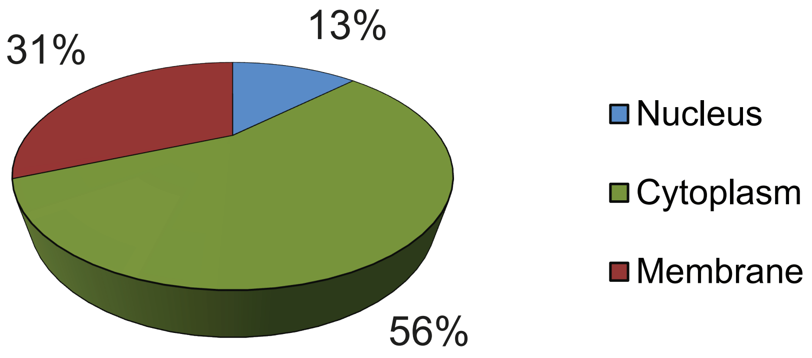


b

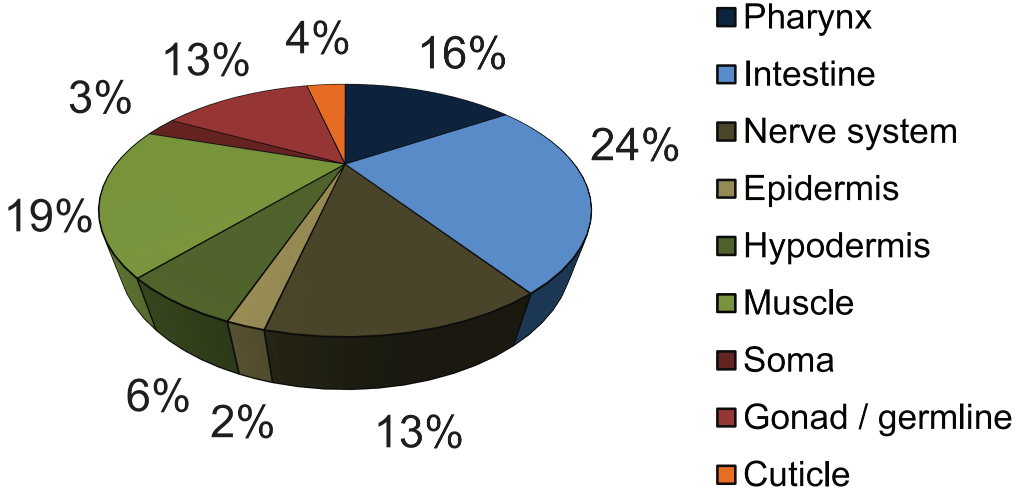

Stout et al, Supplementary Figure S4

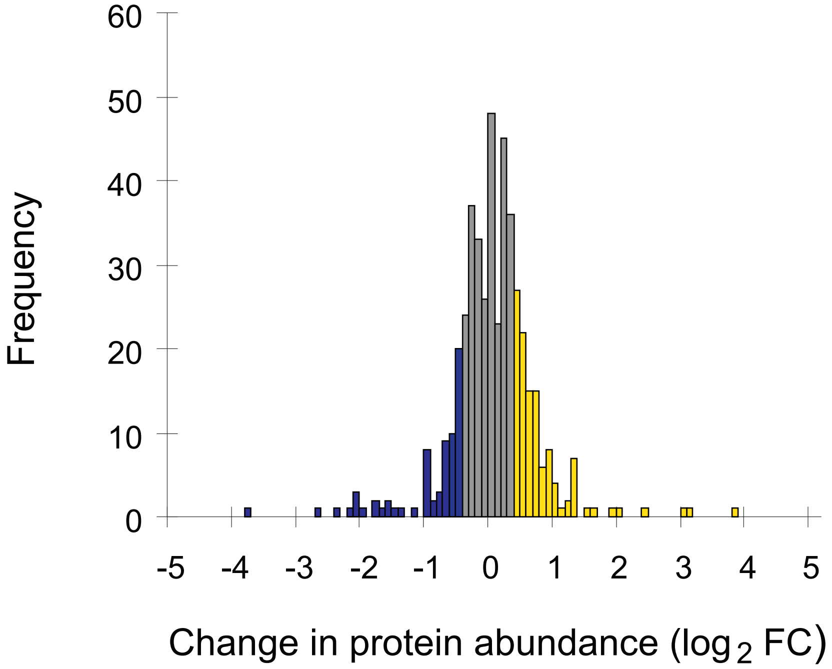

Stout et al, Supplementary Figure S5

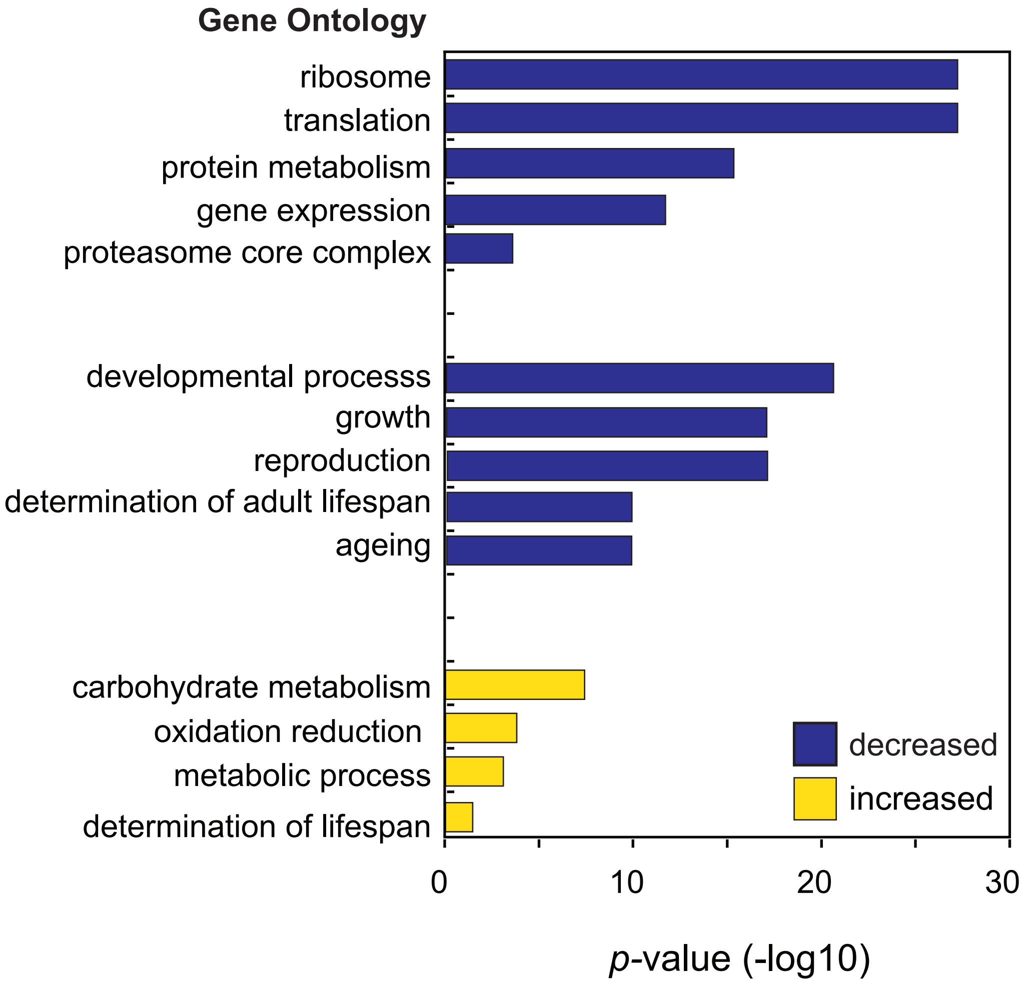

Stout et al, Supplementary Figure S6

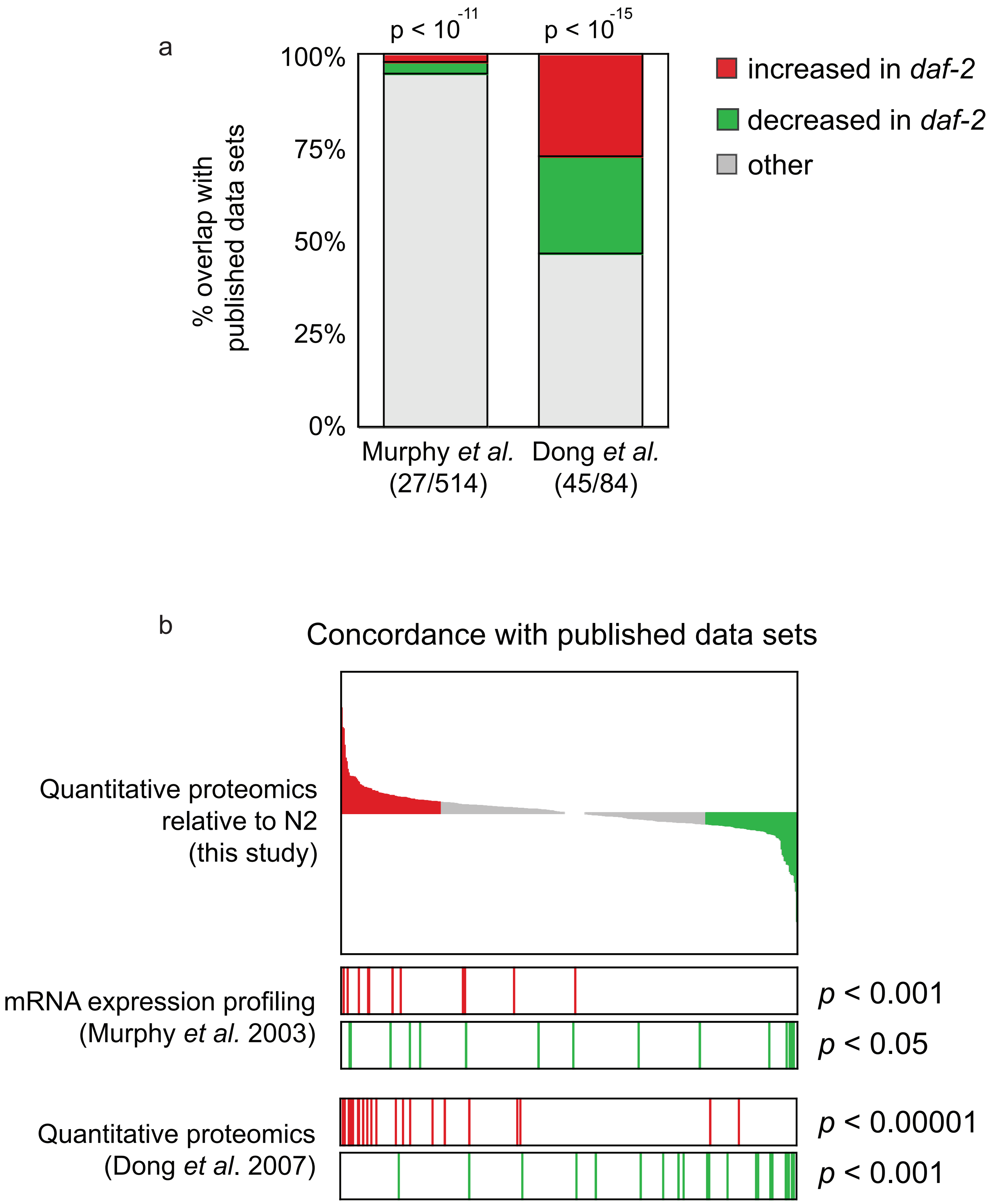

Stout et al, Supplementary Figure S7

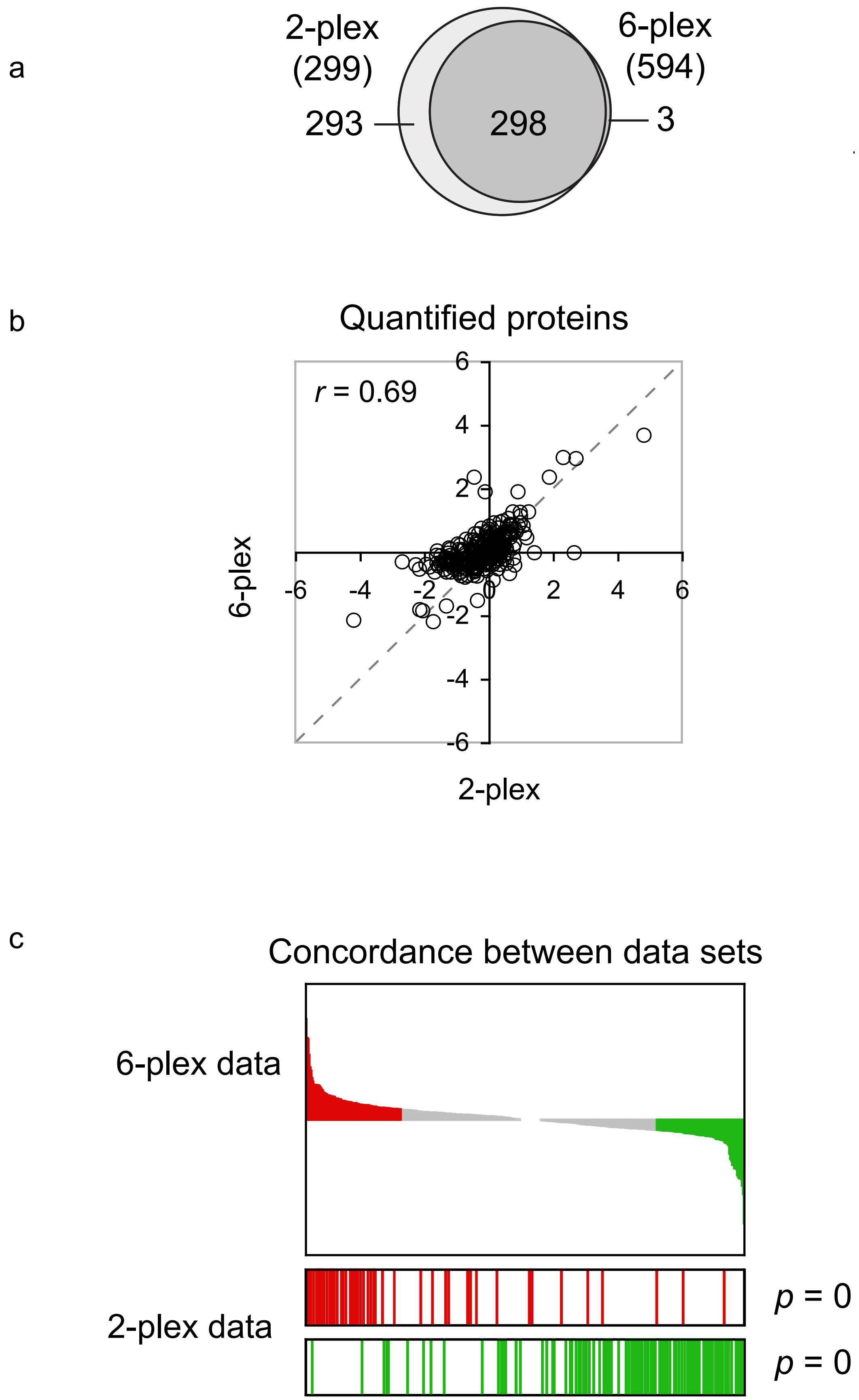

# Stout et al, Supplementary Figure S8

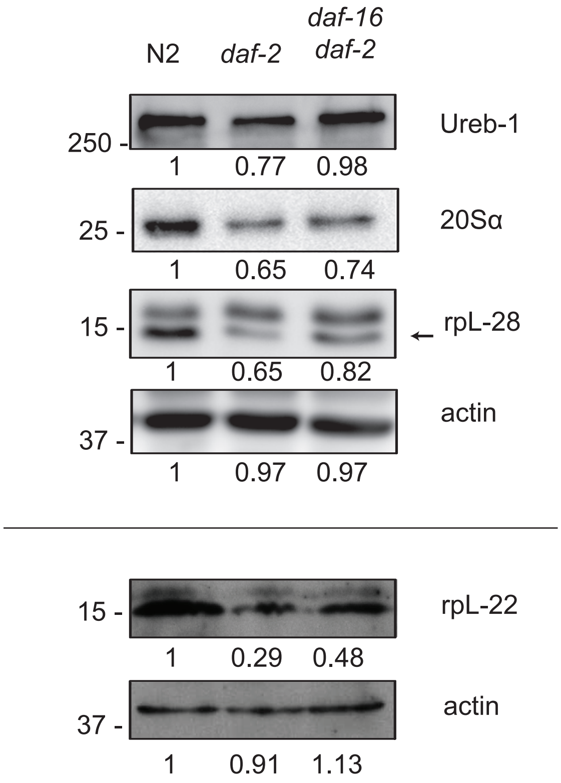

Stout et al, Supplementary Figure S9

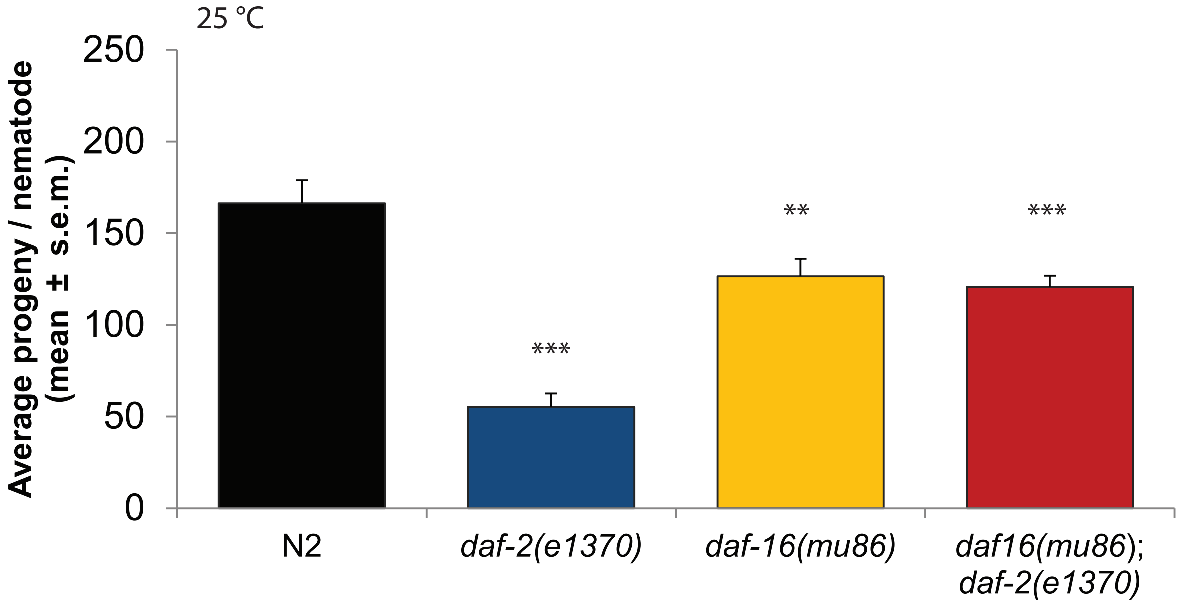

Stout et al, Supplementary Figure S10

a

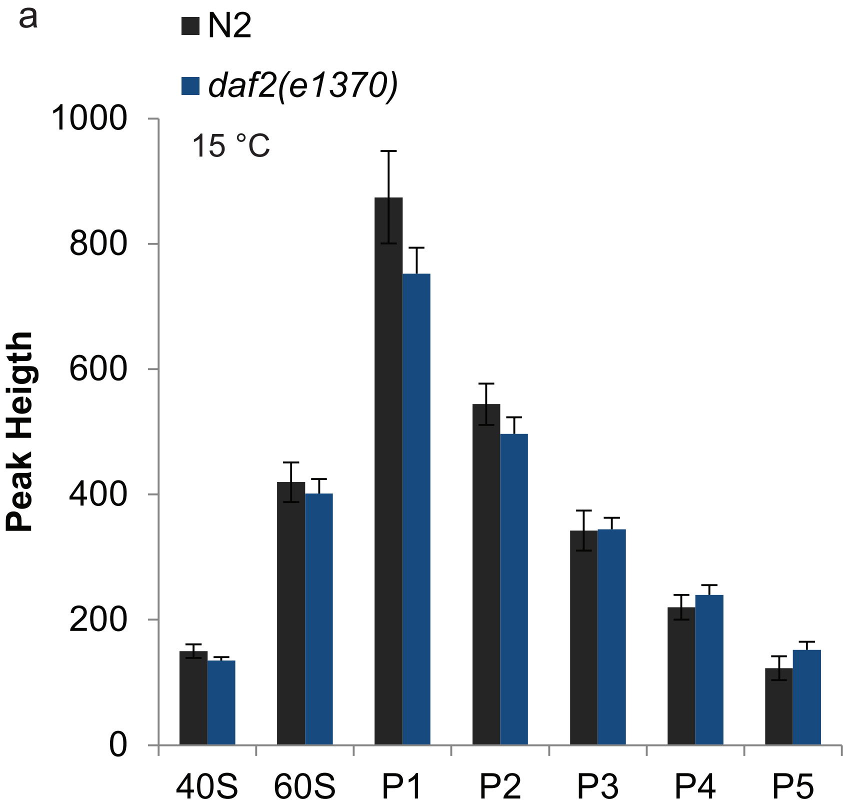


b

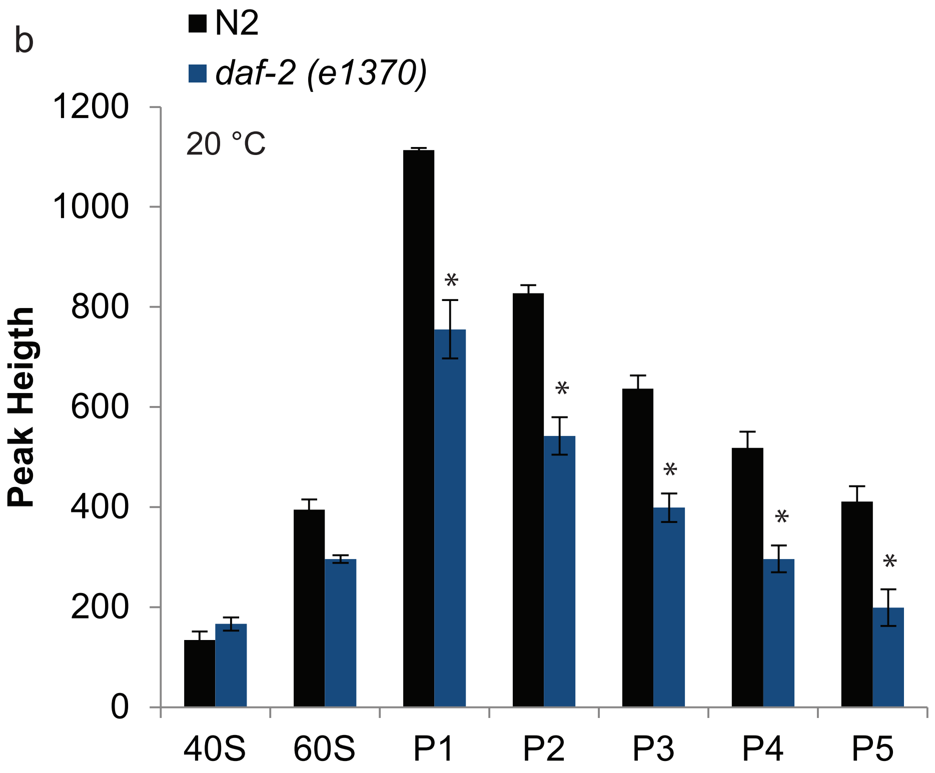

Stout et al, Supplementary Figure S11

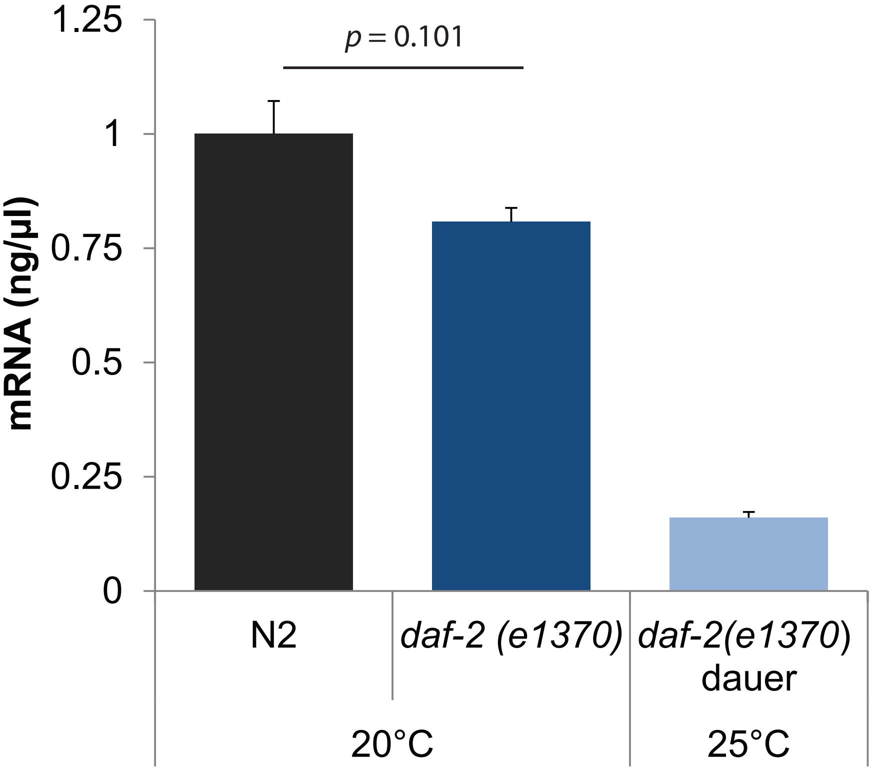

Supplement: Supplementary Information — Supplementary Figures S1–11 [file msb201335-s1.pdf]
